# Retaining doctors in emergency medicine: an ethnographic study of emergency departments in England

**DOI:** 10.1136/bmjopen-2024-086733

**Published:** 2024-09-18

**Authors:** Daniel Darbyshire, Liz Brewster, Rachel Isba, Richard Body, Dawn Goodwin

**Affiliations:** 1 Lancaster University Faculty of Health and Medicine, Lancaster, UK; 2 Manchester Foundation Trust, Royal Manchester Children's Hospital, Manchester, UK; 3 Lancaster Medical School, Lancaster University, Lancaster, UK; 4 Lancaster University, Lancaster, UK; 5 Emergency Department, Manchester University NHS Foundation Trust, Manchester, UK; 6 The University of Manchester, Manchester, UK

**Keywords:** Health policy, QUALITATIVE RESEARCH, Emergency Departments, Physicians, Health Workforce

## Abstract

**Abstract:**

**Objectives:**

To gain a deep understanding of factors driving retention in emergency medicine. To understand in detail the day-to-day lived experience of emergency medicine doctors, to identify and explore factors influencing retention, to situate these descriptions within the current educational and health policy contexts and to advance the debate and make policy and practice recommendations.

**Design:**

Ethnography and semistructured interviews.

**Setting:**

Two purposively sampled emergency departments in England, with additional interview participants recruited via social media and relevant stakeholder organisations.

**Participants:**

41 interview participants comprising 21 emergency physicians across 2 sites, 10 former emergency physicians and 10 stakeholders, with 132 hours of observation over 11 weeks in one emergency department in England.

**Results:**

Three key themes were developed as relevant to the day-to-day lived experience of work in the emergency department, presenting challenges to retention and opportunities for change. First, emergency physicians needed to develop workarounds to mitigate the sensory and material challenges of working in a difficult environment.

Second, education influences retention through valuing, fostering competence and entrustment and supporting interdependence. These were primarily observable in the workplace through senior staff prioritising the education of more junior staff.

Third, community was important for retention. Linked to education through communities of practice, it was built by brief interpersonal interactions between emergency department workers.

Situating these descriptions in current policy contexts identified less than full-time working, portfolio careers and mentorship as retention strategies. Self-rostering and annualisation facilitated these retention strategies.

**Conclusions:**

The emergency department represents a difficult environment with many challenges, yet by focusing on how doctors navigate these difficulties, we can see the way in which retention occurs in everyday practices, and that valuing staff is critical for retention.

**STRENGTHS AND LIMITATIONS OF THIS STUDY**

- 11 weeks of ethnographic fieldwork allowed for a comprehensive sampling of different aspects of emergency medicine work across all areas of the emergency department and all times of day and night.
- Semistructured interviews with emergency physicians of all grades provided a depth and breadth of qualitative data.
- The protocol was published in advance with a clearly described plan for analysis.
-  A second ethnographic site was not feasible due to the COVID-19 pandemic.

## Introduction

 Staff retention is a vital part of workforce planning and service delivery,^1^ but in the UK, doctors are leaving the National Health Service (NHS) in unsustainable numbers.^2^ Healthcare staff retention is a policy priority for the NHS^13^,^5^ and across the globe.^6 7^ The retention problem is particularly acute in emergency medicine and is a research^8^ and policy imperative for the specialty.^9^

Retention is a critical factor in the quality of care as more experienced and senior doctors perform fewer unnecessary tests, make better decisions and get fewer complaints.^10^,^12^ Staffing levels are correlated with mortality across medicine, nursing and allied health professionals, with evidence also suggesting that increased nursing staff seniority reduces inpatient mortality levels.^13 14^ Conversely, high staff turnover is correlated with poor organisational performance^15^,^18^; moreover, it is associated with high financial costs across multiple fields including education,^19^ hospitality^20^ and healthcare.^21 22^ Recruitment can be an effective means to increase staffing levels, but without addressing retention, it is unlikely to be successful in the longer term.^1 3^

A 2021 literature review on retention in emergency medicine found that while up to 35 factors are associated with retention, none predominate and the interplay between factors remains unclear^23^; a finding mirrored in a 2018 review of the broader retention literature.^24^ Flexible working,^25^ peer support,^26^ portfolio careers^26^ and team working^25 27^ were identified in the emergency medicine literature review as being significant factors that aided retention.^23^ Drivers of exodus in emergency medicine^23^ included the physical and emotional strain of the job,^26^ stress,^28^ burn-out^27^,^29^ and poor job satisfaction.^30^ A 2022 ethnographic study highlighted the interplay between factors illustrating how healthcare workers were obliged to prioritise system pressures over personal needs, ultimately driving exodus.^31^

However, understanding the retention is limited by the difficulty in defining it precisely and consistently.^23^ Previous studies have adopted various cut-off points at which it may be said that staff have been retained.^16 32 33^ While a tightly defined time period is necessary for quantitative studies, there remains a sense in which it is arbitrary and unsatisfactory—if staff leave a day after the cut-off point, can they really be said to have been retained? The interplay of factors leading to attrition may be outlined, but there is a lack of clarity about the length of time between experiencing one or more factors and actively leaving. What is missing from current literature is an understanding of what retention looks like in practice on a day-by-day basis, what actions it takes to be retained, and how these actions can be embedded in working environments and teams.

To better situate understandings of retention, we sought a definition and study design that would focus on the ongoing daily achievement of retention, rather than a fixed point at which it is achieved. We adopted a broad definition of retain, the transitive verb, defined as ‘to keep in possession or use’^34^ and retention as ‘the act of retaining’, ‘the power of retaining’ and ‘something retained’.^35^ This definition is helpful as ‘to keep in possession or use’ emphasises the ongoing nature of retention and ‘the act of retaining’ highlights that retention is something that can be done, be done in a particular way and has been done.

Our study, therefore, aimed to gain a deep understanding of retention in emergency medicine and focused on the day-to-day work of doctors in the emergency department. This aim was informed by the lead author’s experience as an emergency medicine clinician and advocate for sustainable working practices and required an ethnographic study design that could account for the complex interplay of factors involved in retention. The objectives were to:

1. Understand in detail the day-to-day lived experience of emergency medicine doctors to identify and explore factors influencing retention.
2. Situate these descriptions within the current educational and health policy contexts.
3. Advance the debate and make policy and practice recommendations based on a detailed understanding of the retention of medical staff in emergency medicine.

## Methods

**Figure 1:**
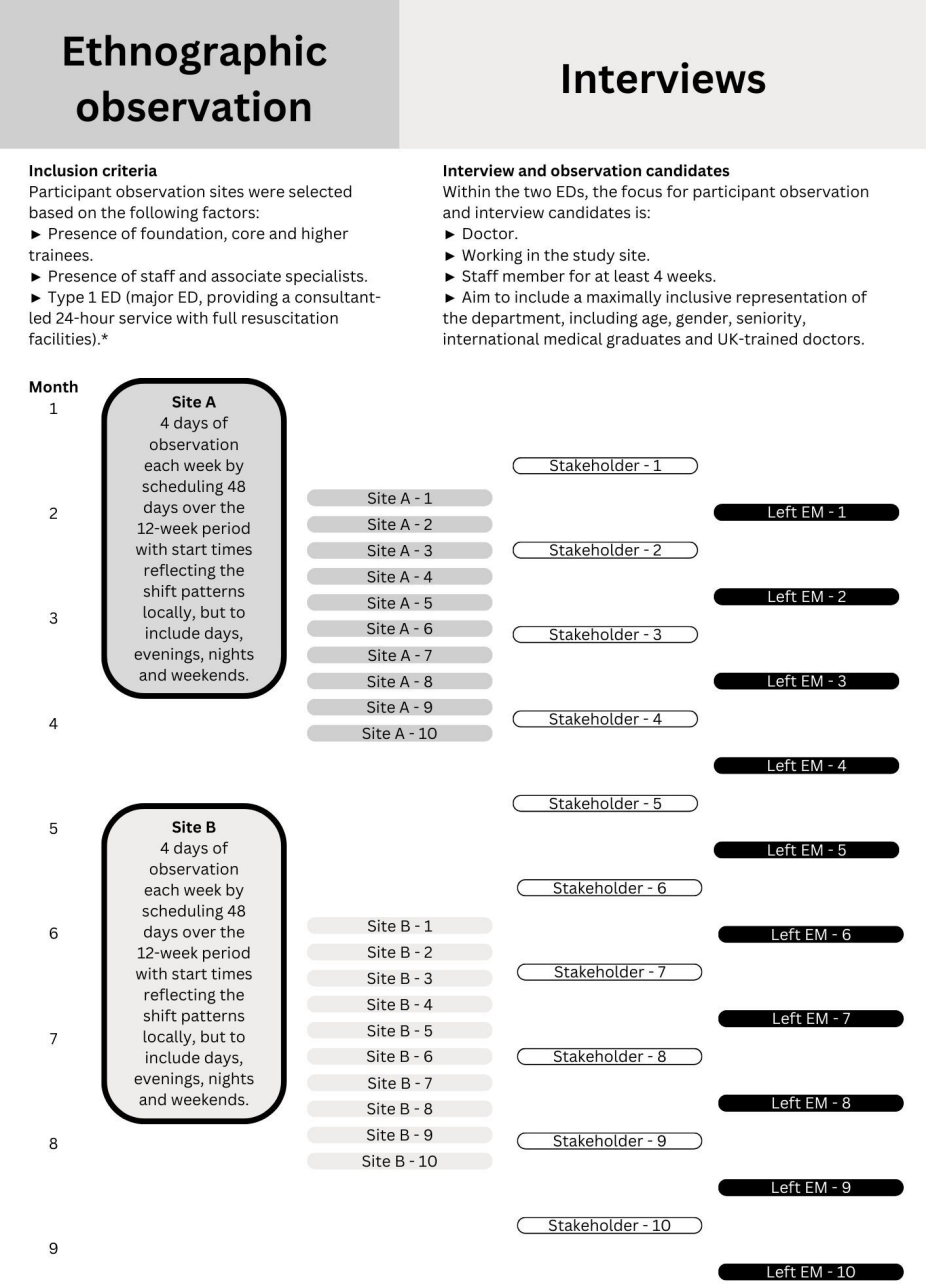
Overview of study protocol. *NHS Data Dictionary. Accident and emergency department type. NHS data model and dictionary version 3, 2018. Available: https:// www.datadictionary.nhs.uk/data_dictionary/attributes/a/acc/ accident_and_emergency_department_type_de.asp (Accessed 5 February 2019). ED, emergency department; EM, emergency medicine.

**Figure 2:**
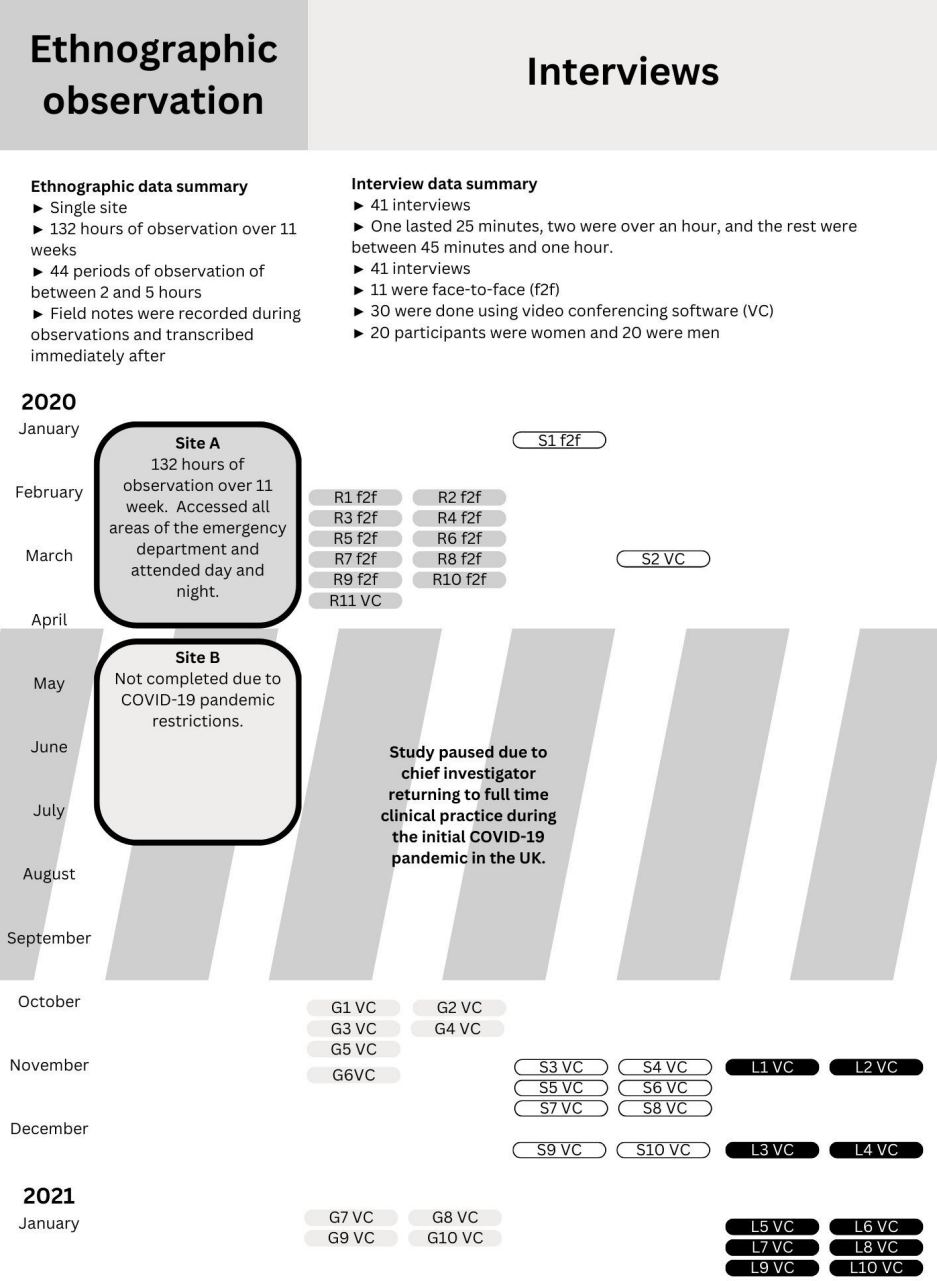
Overview of study data collection as managed due to the SARS-CoV-2/COVID-19 pandemic. The study was conducted from the start of January 2020 to the end of January 2021. There was a 6-month pause in data collection from April to September 2020. f2f refers to face-to-face interview. VC refers to an interview conducted via video conferencing software.

The protocol for this study has been published^36^ (summarised: figure 1) as has a scoping review of the literature on retention of doctors in emergency medicine.^23 37^ The study was interrupted by the SARS-CoV-2/COVID-19 pandemic which necessitated revisions to data collection plans. Consequently, empirical data collection included 41 interviews and 132 hours of ethnographic observation (figure 2).

### Setting and participants

The study was undertaken in the English NHS. We had ethical approval to study two emergency departments (pseudonymised as The Royal and The General) which were purposively selected to encompass the two main distinctions of emergency departments in the UK: a major trauma centre and a district general hospital. Both departments were busy, served an urban area and the surrounding rural population and neither were newly built or redeveloped departments.

Recruitment for interviews was via fieldwork contacts and snowball sampling; through invitations made after potential participants’ relevant local, regional or national role in emergency medicine retention was identified; or via links with the Royal College of Emergency Medicine (RCEM). Recruitment of those who had left emergency medicine was facilitated by word of mouth and social media.

### Data collection: ethnography

DD, an emergency medicine physician, undertook approximately 132 hours of ethnographic observation at The Royal over a period of 11 weeks. Each observation lasted 2–5 hours and covered the full spectrum of work in the emergency department (table 1). Each observation was different but would typically start with DD introducing themselves to shift leaders and staff members working in the area, including giving a summary of the study and often leading to a brief conversation about aspects of retention. DD would spend time in different areas of the department during each observation.

**Table 1:** Summary of fieldwork

Date	Day	Time	Hours observed	Focus of observation period	
				Topics	Locations
7 January	Tuesday	AM	4	1st day, team working, environment.	Sim suite. The Royal.
8 January	Wednesday	PM	3	Natural light, learning, breaks.	Majors area, office space.
10 January	Friday	PM	2	Uniform, facilities for staff.	Corridor and overflow areas.
12 January	Sunday	Late	3	Crowding, staffing.	Majors.
13 January	Monday	AM	4	Handover, exit block, staffing, team, humour.	Borrowed space, majors.
14 January	Tuesday	AM	4	Environment, dark humour.	Paediatric area, seminar room.
17 January	Friday	AM	4	National quality, end of night shift, handover, shift pattern.	Resus, doctors’ office.
22 January	Wednesday	AM	3	Case discussion, dark humour, fans, noise, systems.	Waiting areas, co-ordinators desk.
23 January	Thursday	Night	4	Temperature, atmosphere, chaos.	Doctors’ office, majors.
24 January	Friday	Night	2	Work, new space, interpersonal relationships.	Resus, rapid assessment and treatment (RAT) area.
25 January	Saturday	Late	3	Atmosphere, leadership, objects, teaching, broken machines, bleeping.	Majors, resus.
27 January	Monday	AM	4	Traffic, portfolio careers, respite, atmosphere of calm, teaching.	Journey into the emergency department. Resus, borrowed space.
29 January	Wednesday	AM	3	New and old space, broken equipment, temperature.	RAT area.
30 January	Thursday	Night	4	End of night shift, lighting, movement, exodus from NHS, retention work.	Majors, RAT area, resus.
31 January	Friday	Night	3	Team, difference with flow, broken equipment.	Majors, corridors and ancillary space.
1 February	Saturday	Night	2	Coffee cups and water bottles, referral, conflict, relationships.	Seminar room, majors.
4 February	Tuesday	AM	4	Leadership, working environment, policy, culture, wider NHS.	Education centre.
5 February	Wednesday	Late	3	Rota, staff toilets, scrubs.	Seminar room.
6 February	Thursday	Late	4	Improvisation with equipment.	Majors, borrowed space.
9 February	Sunday	AM	2	Portfolio careers, retirement, blocked space, repurposing space.	Co-ordinators desk, RAT, borrowed space.
10 February	Monday	AM	4	Repeated minor inconvenience, noise, flow, crowding.	Majors, paediatric area.
11 February	Tuesday	PM	4	Atmosphere, trauma call, training.	Resus.
13 February	Thursday	PM	3	Inadequate facilities, rota.	Office space, majors.
14 February	Friday	AM	4	Temperature, noise, broken equipment, handover.	Borrowed space, resus, seminar room.
17 February	Monday	PM	2	Lack of windows, staff sickness.	Office space, borrowed space.
18 February	Tuesday	AM	2	Missing equipment, valuing, ultrasound machine.	Majors.
20 February	Thursday	AM	5	Handover space, air conditioning, navigating the department.	RAT area, seminar room.
22 February	Saturday	PM	2	Decoration in paediatric area, radio, cleaning.	Borrowed space, paediatric area.
25 February	Tuesday	Late	4	Smell, crowding, problems locating equipment, idle chatter.	Moved across all clinical areas.
27 February	Thursday	Late	2	Supervision, broken equipment, roles, rest of hospital in darkness.	Walked to department through adjoining corridors.
29 February	Saturday	AM	2	Humour, narrative.	Resus.
1 March	Sunday	AM	2	Water bottles, teamwork	Majors areas.
2 March	Monday	PM	3	Physical environment, broken and missing equipment.	Seminar room, office space, borrowed space.
3 March	Tuesday	AM	4	Conflict, colleagues, rest facilities, light, sound, atmosphere	Majors areas.
5 March	Thursday	AM	2	Handover, humour, uniform, office space.	Seminar room, office space, majors.
6 March	Friday	AM	3	Education, narrative.	Majors, RAT.
10 March	Tuesday	PM	3	Lack of space, space between trauma call and arrival of patient	Majors, resus.
11 March	Wednesday	PM	2	Breaks, coffee cups and water bottles, noise, lighting, corridor care	Corridors, majors, break room.
12 March	Thursday	Late	3	Chat, humour, brief interactions, noise, uniform.	Majors, borrowed. Space.
13 March	Friday	Late	2	Visiting the space, humour.	Paediatric area, majors.
16 March	Monday	AM	3	Handover space, absence, handwashing, eating in a corridor.	Seminar room, corridor, majors.
17 March	Tuesday	AM	1	Documentation, computers, handover, signs.	Majors.
18 March	Wednesday	Late	3	Staggered shift starts, narrative, scrubs.	RAT, paediatric area.
19 March	Thursday	AM	2	Apprehension, change, team.	Majors, resus.

All data are collected in 2020. AM refers to 7:00 hours to noon, PM to noon to 18:00 hours, late 18:00–23:00 hours, night 23:00–7:00 hours. Hours observed are approximate. The focus of the observation period refers to key topic summaries extracted from field notes. Location refers to prominent parts of the department from field notes. Borrowed space refers to areas that the emergency department has expanded into either permanently or temporarily without being redeveloped.

NHSNational Health Service

Field notes were handwritten and anonymised using pseudonyms. After each period of observation, DD would transcribe and expand on the field notes, documenting analytical and theoretical insights thereby starting the analytical process.

### Data collection: interviews

All interviews were undertaken by DD. 41 interviews were conducted in total (table 2), 21 with emergency physicians (11 from The Royal and 10 from The General), 10 with stakeholders from organisations with interest in the retention of emergency physicians, and 10 with people who had left emergency medicine (interview guides are available as online supplemental file 1 and online supplemental file 2). Interviews with participants from The Royal followed fieldwork and built on observations. Other interviews were independent of fieldwork.

**Table 2:** Interview participants

	Interview number	Grade (The Royal and The General)/role (stakeholder)/final role in emergency medicine (left emergency medicine)	Gender	Interview
The Royal	R1	Doctor in training	Male	Face to face
	R2	SAS/LE doctor	Male	Face to face
	R3	Doctor in training	Male	Face to face
	R4	Doctor in training	Female	Face to face
	R5	Doctor in training	Male	Face to face
	R6	Doctor in training	Male	Face to face
	R7	Specialist	Female	Face to face
	R8	Doctor in training	Female	Face to face
	R9	Specialist	Female	Face to face
	R10	Specialist	Male	Face to face
	R11	Specialist	Male	Video call
The General	G1	Doctor in training	Male	Video call
	G2	Specialist	Male	Video call
	G3	Specialist	Male	Video call
	G4	Specialist	Male	Video call
	G5	SAS/LE doctor	Male	Video call
	G6	Doctor in training	Male	Video call
	G7	SAS/LE doctor	Female	Video call
	G8	Doctor in training	Female	Video call
	G9	SAS/LE doctor	Female	Video call
	G10	SAS/LE doctor	Female	Video call
Stakeholders	S1	SEB clinical fellow	Female	Face to face
	S2	SEB leadership role	Female	Video call
	S3	Academic working with SEB	Male	Video call
	S4	Healthcare campaigner	Male	Video call
	S5	Royal college committee member	Female	Video call
	S6	Clinical psychologist	Female	Video call
	S7	Healthcare systems expert	Male	Video call
	S8	Human resources director	Female	Video call
	S9	Medical education researcher	Female	Video call
	S10	Workforce development project manager	Male	Video call
Left emergency medicine	L1	SAS/LE doctor	Female	Video call
	L2	Doctor in training	Male	Video call
	L3	Doctor in training	Female	Video call
	L4	Doctor in training	Male	Video call
	L5	Doctor in training	Female	Video call
	L6	Specialist	Male	Video call
	L7	Specialist	Female	Video call
	L8	Doctor in training	Female	Video call
	L9	SAS/LE doctor	Male	Video call
	L10	Specialist	Female	Video call

Interviews from The Royal were conducted during February–March 2020. Interviews from The General were conducted during October 2020–January 2021. SAS/LE doctor refers to specialty and specialist doctors and locally employed doctors. Specialist is a consultant-grade doctor. Interviews from organisations with a stakeholder interest in retention were conducted during January–December 2020. Interviews with doctors who had left emergency medicine were conducted during November 2020–January 2021.

SAS/LEstaff and associate specialist/locally employedSEBstatutory educational body

Most interviews lasted 45–60 min, and all were audio recorded and transcribed in full using NVivo transcription.^38^ In addition, DD made notes after each interview, which were incorporated into field notes.

The final four interviews from The General were conducted within a few days of each other. These interviews confirmed we had reached theoretical saturation with practising emergency physicians.^39^ A similar decision was made with stakeholders and those who had left.

### Data collection: COVID-19 adjustments

The SARS-CoV-2/COVID-19 pandemic prevented ethnographic observation from taking place at The General. Interviews were still conducted by moving online and using contacts made in the study development to recruit.

### Analysis

Interview transcripts and field notes were uploaded to NVivo for Mac^40^ and tagged with demographics to facilitate navigation of the dataset. Following this, DD conducted an iterative reflexive thematic analysis.^41^,^43^ Using this process DD generated themes, codes and text describing these items, all held within NVivo to aid organisation of data.

Multiple candidate themes were identified, but the analysis focused on the themes of ‘workarounds’, ‘retention work’ and ‘retention strategies’. Choices about which themes to analyse further were primarily driven by the data and the aims and objectives of the study; for example, the idea of ‘retention strategies’ is strongly linked to the study objectives of advancing debate and making recommendations situated within current educational and health policy contexts. Analytical choices were also influenced by existing literature related to the research aim and objectives, as well as policy documents, such as the RCEM guide to retaining staff.^9^

Reflexivity was a key element of the analysis, interrogating and integrating DD’s perspective on working as an emergency physician and in national roles in organisations including RCEM, alongside their engagement with the environment as an ethnographer. While the impact of the oversimplified insider–outsider continuum on ethnography remains contentious, the study was ‘insider’ research, meaning it was conducted by someone familiar with the setting.^44^

### Patient and public involvement

The public involvement plan is detailed in the protocol.^36^ Public and professional engagement shaped the project’s development to explore retention rather than exodus and directed the early analysis to focus on the day to day.

The results of this work will be disseminated to the public through institutional press release, ensuing news articles, social media and an opinion piece authored by the study’s authors that describe the study’s findings for the public.

## Results

We identified three main themes linked to the aim and objectives of the study. Workarounds refer to how emergency physicians counter the material challenges of the emergency department. Retention work is the day-to-day actions of emergency physicians in the emergency department contributing to retention specifically around education and community. Retention strategies are the means by which emergency physicians manage their careers in terms of sustainable working.

### Workarounds: response to a challenging work environment

Actions that emergency physicians took in response to the physical and sensory challenges they faced working in the emergency department could be termed workarounds. This theme contributes to understanding the day-to-day lived experience of emergency medicine doctors to identify and explore factors influencing retention.

The sensory and material challenges of working in an emergency department were varied in our data. It was observable that while the sensory impacts of the emergency department as a workplace were not individually extraordinary, they were incessant and multiple. The emergency department was often loud, ‘windowless but bright’, and invariably either too hot or too cold. One interview participant described how the environment ‘could quite easily become a source of frustration that would make me not want to come into work’ (R1). Basic needs at work were routinely not met: ‘there’s no staff toilet’ (R1); no changing room—‘that’s where I get changed, in the storeroom’ (R4). This sensory challenge was compounded by crowding.

Missing or malfunctioning equipment was another source of frustration. Staff found it inconvenient and embarrassing to take a piece of equipment to a patient only for it to fail to work. This routine encountering of faulty equipment led to the practice of ‘duct taping’^45^ which refers to a workaround to ‘a problem that ought not to exist.’ Multiple examples were observed, for instance, a clinician using a stethoscope to test reflexes as they could not find a tendon hammer, representing duct taping as a necessary practice to mitigate inadequacies of the working environment.

A further method of mitigation we identified we termed ‘repurposing space.’ As identified by interview participant R1 above, the sensory and material experience of the working environment could be a threat to retention. It is constant and visible evidence that the work of the emergency department is undervalued. Repurposing space is a response to the sensory challenges of the workplace that makes the working environment, and working life of staff, more agreeable in the short term and sustainable long term. Repurposing space could be as simple as moving to another part of the department, but it was more than just a kinetic activity. ‘One of the things I [would] try [was] getting some headspace, so if it all got a bit too much I would go to a quieter part of the department, so maybe go sit in the seminar room and write in there for 10 minutes, or in resus if it wasn't a bomb site’ (L1).

### Retention work

Actions that emergency physicians took as part of their work in the emergency department that directly or indirectly, primarily or inadvertently, influenced retention can be termed retention work. While workarounds relate to problems that ought not to exist, retention work is part of emergency medicine. The theme of retention work was primarily visible through education and community. Education influences retention through valuing, fostering competence and entrustment and supporting interdependence. Community, while linked to education through the concept of communities of practice, was built through brief interpersonal connections which in and of themselves influenced retention. This theme adds further detail to understanding the day-to-day lived experience of emergency medicine and starts to situate the descriptions within policy contexts. We used ‘work’ in the name of the theme to highlight that the observed actions were effortful and part of the milieu of work in the emergency department.

### Education as retention work

**Figure 3:**
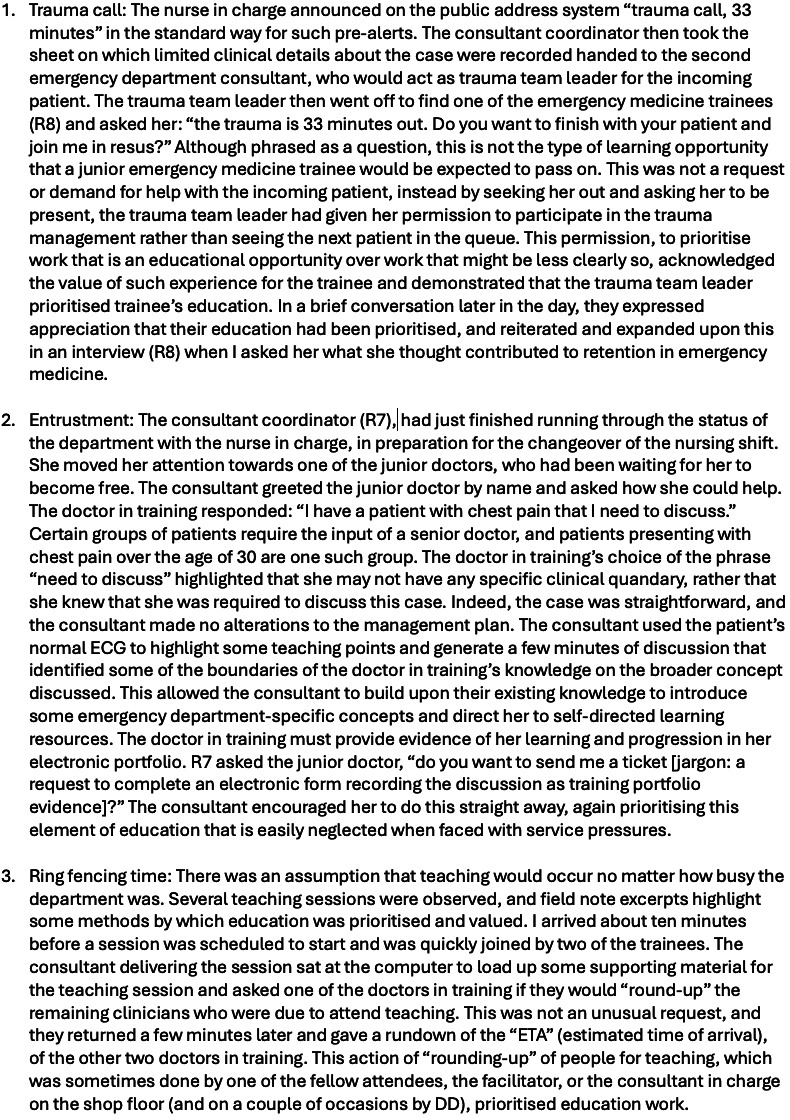
Field note excepts exemplifying prioritising education as a form of retention work.

Education influences retention through valuing, fostering competence and entrustment and supporting interdependence. These forms of retention work were primarily observable in the workplace through senior staff prioritising the education of junior staff. One way that prioritising education manifested was by involving learners in cases where their presence might not be necessary for the clinical management but where they would gain required experience, knowledge, or skills (figure 3, example 1—trauma call). A second way that education was observed as retention work was when a routine case discussion between the senior clinician and those seeking their input was adapted to include an overtly educational element (figure 3, example 2—entrustment). The third way that education was prioritised was the ring-fencing of teaching time (figure 3, example 3—ring-fencing). Figure 3 provides field note excepts exemplifying each way of prioritising education.

The junior doctors in our study were made to feel that their learning was important to the team, by the actions of those leading the team. The value placed on their education as an intrinsic part of their work created space to develop in the emergency department, and they felt this fostered sustainability in their careers. Likewise, for the consultants, these elements of education enriched their interactions with trainees and made a qualitative difference in their daily working lives.

By prioritising time to have meaningful educational conversations, emergency physicians were able to build trust in one another. Trust is an emotive term tied to notions of morality; here we use the neologism ‘entrustment, or ‘entrustability’ to show how education relates to a specific task and not a judgement of character.^46 47^ Our data also highlight the value of education in terms of retention for those doing the teaching by adding variety to their work. Incorporating education involved extending the time taken for interactions, and it made visible the value of caring about one’s colleagues for retention. Participant G4 explained: ‘I like squeezing in bits of teaching … it adds some variety to my shift as well, and I can learn about the trainees as well.’

### Community as retention work

The oft-repeated phrase ‘the practice of emergency medicine is a team sport’^48^ is used to highlight one of the attractive elements of emergency medicine to potential applicants for specialty training. However, we found that the ‘team sport’ analogy did not capture the realities of teamwork in the emergency department and what was important about this teamwork for retention. We instead use the term ‘community’ to describe the interplay between colleagues at an informal level, learning and notions of togetherness: ‘I particularly enjoy working with colleagues and I like that aspect of emergency medicine that … not feeling like you're working in isolation, but you're working with colleagues both from a camaraderie point of view… but also from a learning point of view that you can chat about difficult cases and get someone else’s suggestions on it.’ (R3)

Another trainee developed this idea of camaraderie further: ‘So, I think in terms of the department we are quite a tight knit group, there is a lot of human interaction with the team as well, which I quite enjoy, and I'd say so yeah, that’s kept me going’ (G6). The intensity of human interaction, and the strong bonds signified by a ‘tight-knit’ community is what strengthened their commitment to working in the emergency department. Such talk and interactions were readily visible in the emergency department, but they were brief, which, taken out of context, may be interpreted as unimportant. However, when taken together, they build belonging, and strengthen the community. One participant described the process as ‘repeatedly making new teams’ (R6) reiterating the continual effort required to do the retention work of community.

### Retention strategies

The final theme, retention strategies, involved understanding the contribution of less than full-time working, portfolio careers and mentorship. This theme further develops the policy context, as well as advancing the debate with policy and practice recommendations for improving retention of medical staff in emergency medicine. Doctors working in emergency medicine described three strategies that they believed helped with retention for them and the wider workforce. The first strategy used less than full-time working and portfolio careers to manage the working week. Participants described the impact of the challenging working environment and the ways they sought to mitigate this by limiting the time they spent there. ‘I think the main coping technique now is a portfolio career and not to do emergency medicine full time.’ (A11)

The second retention strategy is related to managing emergency department staff rostering. Participants reported that a good rota helped retention and a bad one hindered it. A common instance was poorly planned transitions from night shift to day shift ‘The rota is, I think, particularly poorly designed, with very quick transitions between night shifts and day shifts and lots of them with very little downtime and an opportunity for recovery.’ (R1) A well-managed rota allowed participants to plan essential parts of their lives such as childcare and family holidays, respond to unexpected events and meet training requirements. Participants recognised that managing an emergency department rota is difficult, but that doing so made them feel valued as members of the team.

The third retention strategy emergency medicine doctors employed relates to mentorship. Participants mainly talked about their ‘mentors’ rather than the process of mentorship, and described a mentor as a ‘trusted counsellor or guide’, in line with the dictionary definition.^49^ The most significant aspect of mentoring for retention was the guidance it provided on managing the demands of a career in emergency medicine: ‘All of the people that I consider to be my role models do something else. So, none of them are full-time generic EM [emergency medicine] people. So, [person] does all his [clinical sub-specialty], and [clinical sub-specialty] is not something that interests me. But I think the balance that he had, you could see makes him happy and is something to then aspire to. [Second person] does the [education] stuff, which got me into the [education] side of things. But again, she was very forthcoming with the fact that it gave her balance and gave her something else to do.’ (R7) Mentors and mentorship, like less than fulltime working and portfolio careers, were ways to manage the working week and careers in the longer term.

## Discussion

In summary, this study delineates what emergency physicians do day to day to continue to work in a challenging specialty and working environment. While earlier studies measured links between numerous factors and constructs related to retention, they failed to elucidate how the many influencing factors were important. We have established what mattered to our participants and provided concrete examples of what retention looks like in emergency medicine; on a day-to-day basis they undertook various forms of retention work, and in the longer term, they strategised about how to structure their careers to make them more sustainable.

We found that emergency physicians required respite from the sensory burden of the space. Workplace education was routinised retention work in the emergency department. Working in the context of a community, while it required effort to build and maintain, was important to participants. Key retention strategies were less than full-time working, portfolio careers, mentorship and a well-designed rota mindful of staff needs. Valuing was a thread that ran throughout several themes. The material challenges evidenced by workarounds are forms of undervaluing whereas education, community and support from retention strategies are valued in action. A useful heuristic for policy-makers is therefore that valuing helps retention.

Strengths of the research include the length of workplace observations, conducted over 11 weeks, which provided ample opportunity to observe a variety of situations. One weakness is that fieldwork was only completed at one site with additional fieldwork curtailed by the SARS-CoV-2/COVID-19 pandemic and infection control restrictions, which could lead to concerns about transferability. It is possible that the fieldwork site was in some significant way atypical, however, one of the benefits of insider research is that DD has experience against which to judge how typical the department was. This gives us confidence that the findings apply more broadly.

Being an insider ethnographer brings further strengths and limitations. An outsider researcher may have gained different insights. Nevertheless, DD’s status as an emergency physician allowed him to draw conclusions directly relevant to the policy domain of emergency medicine. Reflexivity allowed us to move beyond the oversimplification of pros and cons, and to integrate this knowledge into the analysis. Another strength is that throughout the research, DD also conducted work parallel to the study, such as evidence submission to policy-makers, again emphasising the practical implications of this research.^50^

Considering the findings of this paper in relation to what is already known about retention, it is clear that much of the research directly pertaining to retention in emergency medicine takes a very particular methodological approach, seeking to correlate measurable factors with proxies for retention. Many factors including burn-out, attainment and job satisfaction have been found to have a statistically significant influence on retention.^23^ However, this literature lacks depth and context. What these studies do not provide is an idea of the importance of specific factors or the mechanism by which they impact retention. Our study complements previous work by taking a novel approach, asking not why people leave, but what do they do to stay? This focus enabled us to elucidate, in concrete terms, what matters to emergency physicians in their working life and how, despite the challenges, they construct sustainable working practices. The ethnographic methodology adds an important depth of understanding, although in the specific context of emergency medicine. In particular, our study provides detail as to how factors identified in other studies including teamwork,^25 27^ education^2551^,^53^ and portfolio careers^26 53^ function in context. Research from outside of emergency medicine follows a similar pattern in lacking depth or context, but on a larger scale. This study is an example of what can be learnt when research deliberately does not follow a ‘dominant analytical mindset’, understood as how research on a topic utilised similar analytical and theoretical tools as prior research, which in turn stunts the development of understanding.^54^ Our study adds to the small corpus of literature that has deviated from the dominant analytical mindset in both the business and management literature and the health professions literature.

The findings suggest that the working environment is a threat to retention—it needs improving. The role of the environment was highlighted in a report for the General Medical Council that outlined the importance of the working environment for doctors’ well-being and retention^55^ and has been a theme in outputs from emergency medicine-focused organisations^56^,^64^ and the broader NHS.^5865^,^67^ Many things would have to change for the clinical space in the emergency department to improve. Crowding, exit-block, hospital-wide capacity problems and difficulty accessing social care in the community are problems beyond the power of an individual department or even the specialty to resolve. Individual emergency departments can improve aspects that are within their control. Adequate break facilities, a changing room and a staff toilet, spaces for education and handover that are fit-for-purpose, are all achievable and would impact the workplace experience. This list of necessary resources aligns with much of the literature related to the working environment but has had specific attention in the form of campaigns again both specific to emergency medicine^60 62^ and targeted at the broader NHS.^68^,^71^ A 2022 ethnographic study including an NHS acute medical unit, which has similarities to an emergency department, found that misalignments and subsequent adaptations between work-as-imagined and work-as-done were ubiquitous.^72^ This study focused on quality and safety but resilient healthcare-informed research may be useful to develop an understanding of retention further.

Prioritising education is important for retention. Both the time and financial resources dedicated to education in healthcare should be increased. The interpretation of the ‘cost’ of education should be reconceptualised; education is an investment in the retention of the current and future workforce. Arguments for prioritising education in healthcare come from many of the different stakeholders including universities^73^ and NHS agencies.^74^ Research from within emergency medicine has started to link the importance of education with retention, but only for certain groups such as established consultants providing the clinical educator role.^75^ Incorporating knowledge about what facilitates retention at all levels provides an additional argument in favour of prioritising education in emergency medicine and across healthcare.

Flexibility in terms of working patterns is achievable in emergency medicine^66 76^ but the contrary, fixed patterns with problems such as rapid changes from day to night-shift working, remain commonplace. There is plentiful data for the physiological^77 78^ and performance harms of poor working patterns^79 80^ that are clearly recognised in several policy spheres,^81 82^ yet remain a significant feature of working life for emergency medicine doctors. Departments struggling to implement self-rostering and annualisation, less than full-time working, and portfolio careers should be offered support. Existing guidance from RCEM should be supported with funding to buy out time for clinicians and managers to develop flexibility locally. This could follow a similar arrangement to previous programmes piloted in emergency medicine.^75 83^

Previously recruitment to emergency medicine has struggled, but this has not been the case for several years now, with successful campaigns from RCEM and statutory educational bodies to improve the image and appeal of the specialty being posited as causative for this trend. Medical students and foundation doctors have also had increased exposure to the specialty. Whether initiatives to improve recruitment have had unplanned consequences on retention is a question that is difficult to address but warrants attention to inform any future alterations to how doctors are encouraged to consider and be selected for specialty training. The implications of the findings of this paper show that it is not enough to fund initiatives to encourage people to join the specialty; it is also vital to understand what makes them stay to ensure that investment in the clinical workforce is sustained over time.

## supplementary material

10.1136/bmjopen-2024-086733 online supplemental file 1

10.1136/bmjopen-2024-086733 online supplemental file 2

## Data Availability

Data are available on reasonable request.
