# Supplementary material for: Retaining doctors in emergency medicine: an ethnographic study of emergency departments in England
Source: BMJ Open. 2024 Sep 18;14(9):e086733. doi: 10.1136/bmjopen-2024-086733 (PMC11418526; doi:10.1136/bmjopen-2024-086733)
Supplement: online supplemental file 1 [file bmjopen-14-9-s001.pdf]

Lancaster University

Interview Guide

Where have all the doctors gone? An ethnographic study to explore the critical retention problem in Emergency Medicine

This guide will be used as a structure for interviews. It will allow the interviewer to draw out value from an interview and a structure to return to if the interview is going off topic. It will be shaped in response to specific observations in the fieldwork and tailored to the individual, with a focus on the overall research question: what makes doctors stay working in emergency medicine?

Pre-interview Checks

Ensure you have completed the consent procedure as outlined in the protocol.

Thank the participant for contributing.

Check the voice recording equipment is working.

Opening Question

To start the interview of ask some background questions, these will vary depending on your pre-existing knowledge of the participant.

- What is your role?
- How long have you worked in emergency medicine?
- What made you decide to work in emergency medicine?

This may direct the interview into retention, if not move onto directly asking about it.

What keeps you working in emergency medicine?

This is a very open question, follow up questions will depend upon the trajectory of the interview but may include:

Can you talk about a specific experience?
Prompts: what affected your decision? What happened after that experience?

Question Relating to Culture

How would you describe the culture of this emergency medicine department?
Is the culture here different to other EDs you have worked in?

Do you think that workplace culture affects staff retention?
How has it affected your work in emergency medicine?

Again, an open question, while generalities about retention and culture are interesting it is important as an interviewer to try and get to the personal experience.

Question Relating to Environment

How would you describe the work environment of this emergency medicine department?
Is the workplace here different to other EDs you have worked in?

How does the work environment affect your decision to work in EM?

Again, open questions, while generalities about retention and work environment are interesting it is important as an interviewer to try and get to the personal experience.

Digging for Depth

Having opened the conversation and grounded it in the interviewee's lived experience related to retention, the interviewer now needs to try and expand and develop any idea. Three tools are available for this:

1. *Being sensitive to ques from the previous questions and returning to them.*
2. *Asking about colleagues' experiences, building on specifics, and then returning to personal views about this.*
3. *Using a social media post to generate further discussion.*

This list is not exhaustive, nor it is a checklist. Some interviews may use all, some none, they are simply options that a skilled and attentive interviewer can apply to try and gain as much value from the interaction.

Post Interview Checklist

On completion of the interview a number of things need to be completed:

- Ensure the interviewee is alright, it is possible that the interview covered some difficult ground, the participant information sheet has sources of support.
- Thank participant for contributing.
- Are you ok? Remember the sources of support are available to the interviewer as well.
- Interviewees can be given a certificate of participation to include in their portfolio, taking part in research as a participant can help clinicians understand the process. This also acts as a formal thank you to participants.

Once the interviewee has vacated the interview space the interviewer should complete field notes related to the interview, the closer to the interview the better.
