# Supplementary material for: Retaining doctors in emergency medicine: an ethnographic study of emergency departments in England
Source: BMJ Open. 2024 Sep 18;14(9):e086733. doi: 10.1136/bmjopen-2024-086733 (PMC11418526; doi:10.1136/bmjopen-2024-086733)
Supplement: Supplementary file 3 [file bmjopen-2024-086733.reviewer_comments.pdf]

PEER REVIEW HISTORY

BMJ Open publishes all reviews undertaken for accepted manuscripts. Reviewers are asked to complete a checklist review form and are provided with free text boxes to elaborate on their assessment. These free text comments are reproduced below.

ARTICLE DETAILS

**TITLE (PROVISIONAL)**	Retaining doctors in emergency medicine: An ethnographic study of emergency departments in England
**AUTHORS**	Darbyshire, Daniel; Brewster, Liz; Isba, Rachel; Body, Richard; Goodwin, Dawn

VERSION 1 - REVIEW

**REVIEWER NAME**	Sanford, Natalie
**REVIEWER AFFILIATION**	KCL
**REVIEWER CONFLICT OF INTEREST**	None to declare
**DATE REVIEW RETURNED**	23-Apr-2024

**GENERAL COMMENTS**	Overall comments: Thank you for the opportunity to review this manuscript! I think that this paper has a lot of potential to contribute meaningfully to the literature on the healthcare workforce and retention. However, I suggest the authors reorganise throughout to make their contributions more clear. Some minor changes are suggested for the abstract and methods to enhance clarity and style. Major revisions are required in other sections, particularly in the results and discussion, to better match the research methods/objectives. To be suitable for publication, the authors should better describe what they observed during the ethnography and provide richer insight into the lived experience of ED clinicians as described in the interviews to align with their objectives and methods.<br><br>Abstract: Please make clearer that data were collected in two hospitals that were purposively selected (and maybe also some other interview participants recruited via social media who weren't affiliated with either hospital? Not clear from methods section. Needs revision). Results: Suggest rephrasing to more directly match the research question/objectives- you currently start by listing things that challenge, rather than support, retention. I think the order could be improved. Conclusions- Could be made less general than at present, both to better align with your paper and to pack more of a punch!<br><br>Intro: Currently feels a little list-y, I think you could better indicate the problem area. Suggested content: P1) Staff retention is a vital part of workforce planning, but doctors are leaving the NHS; end paragraph with the aims of this paper P2) Why retention is important (workforce dilution, patient outcomes, safety, costs etc) See Zaranko et al. 2023- this example is from nursing but still relevant. Peter Griffiths also has some wonderful papers. I am sure there are similar examples in medicine that you can find that empirically demonstrate the importance of adequate staffing and an experienced workforce. You have a bit of this already, but the points

	could be articulated more clearly to highlight exactly why this is important to study. It's also worth mentioning that recruitment efforts have increased but you can't fill a leaking bucket- UK staff are leaving to go to Aus, etc. This is not a recruitment problem (right now this is mentioned a bit in your discussion, but it should also come in here to justify your study). P3) What do we already know about why people are leaving? Cite your review here and any particularly important studies you found. Could also mention that this is worse now because of the pandemic, that healthcare workers are under tremendous pressure (see Sanford et al., 2022 on pressures, for example). You can add your definition of retention here. I think most of your definitions of retention are from the dictionary- it may be more appropriate to take them from healthcare lit. P4) Do we know anything about this in emergency medicine specifically? Why is this a fruitful/interesting/important setting for exploration? P5) Overall- what is missing? What don't we know that we need to in order to solve this problem? Lead into concluding the intro with your current last intro paragraph.<br><br>Methods: Suggest reorganising into the following headings to make more clear after your first paragraph- setting and participants (what you actually did- see further advice above in abstract section), data collection (2 subheadings, ethnography and interviews- what you actually did), data analysis, covid-19 adjustments (what you intended to do in protocol and how that differs from what you did), PPI. In analysis section, edit current phrasing to make it less awkward. The process didn't generate codes- you did via the process. It's okay to say that! Make it more clear how you identified relevant text for each theme. What is retention work and how is that different from a retention strategy? How did you come up with these themes? Also, what do you mean be 'strength of the data?' More detail/clarity is required in this section, though overall I think the content is fine. A few examples of interview questions in text would be helpful to better indicate your area of inquiry.<br><br>Results: Most of the explanation at the start should be re-worked into the methods. Start the results by restating your aims and providing an introduction/road map to the organisation of the results. Explain each theme within its own section.<br><br>In each section, I think you could more clearly explain the overall theme before giving examples. Each needs to also be related more clearly to the topic of retention- I felt I was having to do the mental work of connecting the issues you describe to the impact on staff and therefore retention, and as in the abstract, you start by describing things that (presumably) negatively impact retention (e.g., poor work environment). I think it's fine to do that if that's what people said when you talked to them, but perhaps it needs to be more explicit that these are barriers to retention and maybe this theme shouldn't come first. All three sections could be much richer- they are currently very list-y. What did you observe? What was the environment like? What did staff say about their lived experiences and how these impact their desire to stay or go? Rich narratives would be more in line with your methods. The second and third sections are a bit better, but still require better organisation, theme description, and richer narratives including data from both the observations and interviews. You also have some discussion throughout the results with references to outside lit which I found confusing- I suggest moving the discussion of outside lit to the discussion section and just presenting what you found in your study

	in the results section, with explanation of what it means. Overall, this section requires major revisions<br><br>Discussion: Suggest reordering again here. Highlight your key results, tell us what they mean and how they relate to the wider literature-- what do you add?, provide strengths and limitations, then implications and conclusions. In your current discussion of the lit, you say that your study adds by highlighting why people stay rather than why they go- but actually, in its current form your first results paragraph mostly describes why people go. If you want the reasons people stay to be more salient/the stand-out part of your work, you need to rework your results to reflect that as suggested previously. Also, please note, the purpose of qualitative research is not generalisability, so I would edit that paragraph to reflect the value you are adding with this type of study and how it complements other approaches. I also suggest reading a bit about resilient healthcare as it is quite relevant to the adaptations you describe (e.g., stethoscope as a hammer workaround) and discussing the long-term implications of people compensating for poor work system design. Janet Anderson has many relevant papers on this in healthcare. The implications of your work need to be more explicit- what are you recommending that is different from what others have said?<br><br>I hope you find these comments helpful and best of luck with your submission! :-)

**REVIEWER NAME**	Zhao, Yingxix
**REVIEWER AFFILIATION**	University of Oxford Medical Sciences Division, Nuffield Department of Medicine
**REVIEWER CONFLICT OF INTEREST**	I do not have any competing interest
**DATE REVIEW RETURNED**	29-Apr-2024

**GENERAL COMMENTS**	Thank you for the opportunity to review this manuscript. The paper itself is well written, and the topic is timely relevant considering the growing precarity of NHS workforce. I only have a few comments that hopefully could further strengthen this paper.<br><br>1. I appreciate Table 1 and 2 provided an in-depth overview of data collection processes, however they are relatively long and not contributing much to the reading (and also really long). I recommend putting these into the supplementary appendices and keep the figures in the full text only.<br><br>2. It would be useful to further elaborate the relationship between the observation and the interview. Were they conducted sequentially? Did the initial observation inform your interview questions? Also missing from the appendices is the interview guide if it has been used.<br><br>3. I enjoy reading the reflexivity sections both in the methods and discussion paragraphs. It would be useful to also reflect on the role of all other co-authors, at what stages were they involved and are they also 'insider' or more 'outsider' which could be useful for sense-making.

	4. For the discussion section it would be useful to consider if your findings align with any theories on retention or broadly HRM. For example job demand - control, social cognitive career theory, etc. Having a paragraph discussing relevance to social theory and potential advancement would further strengthen this paper.

VERSION 1 – AUTHOR RESPONSE

Reviewer: 1

Dr. Natalie Sanford, KCL

Comments to the Author:

Overall comments: Thank you for the opportunity to review this manuscript! I think that this paper has a lot of potential to contribute meaningfully to the literature on the healthcare workforce and retention. However, I suggest the authors reorganise throughout to make their contributions more clear. Some minor changes are suggested for the abstract and methods to enhance clarity and style. Major revisions are required in other sections, particularly in the results and discussion, to better match the research methods/objectives. To be suitable for publication, the authors should better describe what they observed during the ethnography and provide richer insight into the lived experience of ED clinicians as described in the interviews to align with their objectives and methods.

Thank you for the constructive feedback. We have responded to each underneath the appropriate paragraph and signposted where changes have been made in the manuscript.

Abstract: Please make clearer that data were collected in two hospitals that were purposively selected (and maybe also some other interview participants recruited via social media who weren't affiliated with either hospital? Not clear from methods section. Needs revision). Results: Suggest rephrasing to more directly match the research question/objectives- you currently start by listing things that challenge, rather than support, retention. I think the order could be improved. Conclusions- Could be made less general than at present, both to better align with your paper and to pack more of a punch!

The abstract has been edited as suggested.

Intro: Currently feels a little list-y, I think you could better indicate the problem area. Suggested content: P1) Staff retention is a vital part of workforce planning, but doctors are leaving the NHS; end paragraph with the aims of this paper P2) Why retention is important (workforce dilution, patient outcomes, safety, costs etc) See Zaranko et al. 2023- this example is from nursing but still relevant. Peter Griffiths also has some wonderful papers. I am sure there are similar examples in medicine that you can find that empirically demonstrate the importance of adequate staffing and an experienced workforce. You have a bit of this already, but the points could be articulated more clearly to highlight exactly why this is important to study. It's also worth mentioning that recruitment efforts have increased but you can't fill a leaking bucket- UK staff are leaving to go to Aus, etc. This is not a recruitment problem (right now this is mentioned a bit in your discussion, but it should also come in here to justify your study). P3) What do we already know about why people are leaving? Cite your review here and any particularly important studies you found. Could also mention that this is worse

now because of the pandemic, that healthcare workers are under tremendous pressure (see Sanford et al., 2022 on pressures, for example). You can add your definition of retention here. I think most of your definitions of retention are from the dictionary- it may be more appropriate to take them from healthcare lit. P4) Do we know anything about this in emergency medicine specifically? Why is this a fruitful/interesting/important setting for exploration? P5) Overall- what is missing? What don't we know that we need to in order to solve this problem? Lead into concluding the intro with your current last intro paragraph.

Thank you for the detailed feedback on the introduction.

We have:

- reordered and restructured as suggested

- added in references as suggested

- noted that the impact of the pandemic would require more space than this paper allows, so we have not been able to address this suggestion.

- maintained the position of the aims/ overview as this aligns with the journal preferred style

- added a sentence about what is missing from existing literature and explained why the dictionary definition is our preferred definition, but there is not scope to fully explore the deficiencies of the definitions used in the healthcare literature in this paper. This is addressed in the referenced papers to a degree.

Methods: Suggest reorganising into the following headings to make more clear after your first paragraph- setting and participants (what you actually did- see further advice above in abstract section), data collection (2 subheadings, ethnography and interviews- what you actually did), data analysis, covid-19 adjustments (what you intended to do in protocol and how that differs from what you did), PPI. In analysis section, edit current phrasing to make it less awkward. The process didn't generate codes- you did via the process. It's okay to say that! Make it more clear how you identified relevant text for each theme. What is retention work and how is that different from a retention strategy? How did you come up with these themes? Also, what do you mean be 'strength of the data?' More detail/clarity is required in this section, though overall I think the content is fine. A few examples of interview questions in text would be helpful to better indicate your area of inquiry.

We have restructured the section and incorporated more detail on recruitment here rather than in the abstract. We have also made it more explicit that the sites were purposively selected.

In the data analysis section we have edited the third sentence related to generation of themes to make it clear that DD generated them not the reflective thematic analysis. We have edited to provide greater clarity rather than talking about the ‘strength of the data’. Rather than include interview questions in the text we have included theinterview guides as supplemental files as requested by the editor.

Results: Most of the explanation at the start should be re-worked into the methods. Start the results by restating your aims and providing an introduction/road map to the organisation of the results. Explain each theme within its own section.

In each section, I think you could more clearly explain the overall theme before giving examples. Each needs to also be related more clearly to the topic of retention- I felt I was having to do the mental work of connecting the issues you describe to the impact on staff and therefore retention, and as in the abstract, you start by describing things that (presumably) negatively impact retention (e.g., poor work environment). I think it's fine to do that if that's what people said when you talked to them, but perhaps it needs to be more explicit that these are barriers to retention and maybe this theme shouldn't come first. All three sections could be much richer- they are currently very list-y. What did you observe? What was the environment like? What did staff say about their lived experiences and how these impact their desire to stay or go? Rich narratives would be more in line with your methods. The second and third sections are a bit better, but still require better organisation, theme description, and richer narratives including data from both the observations and interviews. You also have some discussion throughout the results with references to outside lit which I found confusing- I suggest moving the discussion of outside lit to the discussion section and just presenting what you found in your study in the results section, with explanation of what it means. Overall, this section requires major revisions

The introductory paragraph to the results section has been rewritten with the aim for greater clarity and to be more concise. Rather than moving the opening paragraph to the methods we have cut significantly from the length and focused on providing a scaffold for the sections to follow. The aim of this paragraph is to link the results to follow with the aim and objectives of the study.

In the materialities section we aim to have clarified the link to retention and the rationale for describing some of the drivers for exodus here.

In the retention work of education section, we believe we already make the link to retention sufficiently clearly and use references only to clarify where we refer to concepts and definitions, rather than engaging with the literature as per a discussion. In an attempt to increase the tick description available to the reader, without compromising the flow and length of the article we have created a figure with 3 field note excerpts along a similar line. To this BMJ paper (https://doi.org/10.1136/bmj.d6788). We will be guided by the editor if this is acceptable in its current form or if, for example, it is better as a supplemental appendix.

In the retention work of community section we believe the link to retention is made in the opening paragraph and while the section does not contain rich extracts of observational data the interview excerpts included work well to support the theme.

In the retention strategies section we have moved some of the content to the discussion as recommended and added some further data extracts to support points.

With regard to including richer narratives we aimed to strike a balance between being succinct and providing enough evidence to support our findings. We believe that for this journal erring on the side of brevity is more appropriate. Reproducing thicker descriptions and longer interview extracts would significantly increase the length of the manuscript.

Discussion: Suggest reordering again here. Highlight your key results, tell us what they mean and how they relate to the wider literature-- what do you add?, provide strengths and limitations, then implications and conclusions. In your current discussion of the lit, you say that your study adds by highlighting why people stay rather than why they go- but actually, in its current form your first results paragraph mostly describes why people go. If you want the reasons people stay to be more salient/the stand-out part of your work, you need to rework your results to reflect that as suggested previously. Also, please note, the purpose of qualitative research is not generalisability, so I would edit that paragraph to reflect the value you are adding with this type of study and how it complements other approaches. I also suggest reading a bit about resilient healthcare as it is quite relevant to the adaptations you describe (e.g., stethoscope as a hammer workaround) and discussing the long-term implications of people compensating for poor work system design. Janet Anderson has many relevant papers on this in healthcare. The implications of your work need to be more explicit- what are you recommending that is different from what others have said?

We have kept much of the content of the first paragraph the same but developed the structure and reiterated the context of the aim of the paper. The first paragraph has been split so that the summary of the study is in context of the gap in the literature. The summary of results is then better scaffolded. The paragraph on valuing has then been incorporated as we felt that with the restructuring it better fitted here.

We would be happy to move the strengths and limitations section as suggested but have, at this point, left it where specified by the journal’s author guidance.

We have changed the wording around generalisability to transferability in light of your suggestion.

We have incorporated a paper that builds on Anderson’s work in the discussion using it to describe the intersections and then suggest that it may be a useful place for future work.

The final comment about what are we recommending that is different is a common challenge. Policy makers, other researchers, practitioners, and ourselves, want new answers but this data suggests that we probably just need to do what should already be being done.

I hope you find these comments helpful and best of luck with your submission! :-)

Reviewer: 2

Mr. Yingxi Zhao, University of Oxford Medical Sciences Division

Comments to the Author:

Thank you for the opportunity to review this manuscript. The paper itself is well written, and the topic is timely relevant considering the growing precarity of NHS workforce. I only have a few comments that hopefully could further strengthen this paper.

1. I appreciate Table 1 and 2 provided an in-depth overview of data collection processes, however they are relatively long and not contributing much to the reading (and also really long). I recommend putting these into the supplementary appendices and keep the figures in the full text only.

We have edited table 2 in relation to some editorial comments, it is now slightly shorter but not to the point of truly addressing your concern. We have no preference as to whether the table is in the main body or an appendix and will be guided by the editor

2. It would be useful to further elaborate the relationship between the observation and the interview. Were they conducted sequentially? Did the initial observation inform your interview questions? Also missing from the appendices is the interview guide if it has been used.

We have briefly addressed the relationship between field work and interviews and have added the reference to the interview guide which will be available as a supplementary appendix.

3. I enjoy reading the reflexivity sections both in the methods and discussion paragraphs. It would be useful to also reflect on the role of all other co-authors, at what stages were they involved and are they also 'insider' or more 'outsider' which could be useful for sense-making.

We have tried writing this succinctly and found that it does not work well. Given the ethnographic methods the need for the leads author’s reflexive commentary is clear, however the value for other authors while not insignificant, is perhaps not great enough to warrant the space required to do this section justice. As such we have opted not to add this to the manuscript.

4. For the discussion section it would be useful to consider if your findings align with any theories on retention or broadly HRM. For example job demand - control, social cognitive career theory, etc. Having a paragraph discussing relevance to social theory and potential advancement would further strengthen this paper.

We have added a few sentences reflecting some work on resilient healthcare informed research which has parallels to some of these bodies of work.

VERSION 2 – REVIEW

**REVIEWER NAME**	Sanford, Natalie
**REVIEWER AFFILIATION**	KCL
**REVIEWER CONFLICT OF INTEREST**	11-Jul-2024
**DATE REVIEW RETURNED**	None to declare

**GENERAL COMMENTS**	Many thanks to the authors for this revised manuscript. Significant improvements have been made to the abstract and methods sections in response to the reviewer and editor comments. The added supplementary materials are also very helpful. I still feel that additional revision is required prior to publication, as follows:<br><br>Introduction: Although there have been substantial improvements to the introduction, I still feel that the argument/justification for the research could be enhanced and presented more clearly. There are some sections that remain particularly unclear- for instance, what do the authors mean by 'retain' referring to a place as well as an action? As the authors point out in their response letter, there is a limited word count, but I think the points could still be articulated more clearly whilst maintaining the same succinctness. If necessary, I would cut words later on (about insider/outsider positionality, (or make this more succinct/one sentence long); about interview length (could be added to table), or by focusing the discussion) to enable additional insightful discussion of the literature in both the introduction and discussion sections of the manuscript.<br><br>Methods: The methods section of the paper is much improved. A few comments: What is meant by conceptual rigour? The concept of data saturation is quite contentious in qualitative research; I would delete this sentence. Please ensure consistent verb tense throughout the methods section. Additional detail has been added to both the data collection and analysis sections, which has enhanced methodological consistency and transparency.<br><br>Results and Discussion: The authors have incorporated a new sentence or two at the start of each results section which is helpful in better explaining the themes. Still, I find the 'themes' confusing and inconsistent- it seems like they are micro/meso/macro influences of retention (though not discussed as such) and I find the word choice of 'materialities' 'work' and 'strategies' of retention confusing. The first theme, materialities, seems to refer to the on the ground decisions team members make/work arounds they use to mitigate the impact of their work environment? As discussed in my original feedback, it seems to me that these workarounds, whilst propping up the system, would also indicate a need for work-system re-design and may actually threaten retention. The authors also make this argument to a certain extent, which is great, but to me the problem is that this theme seems to directly contrast with their aims/assertion that they make early on in the paper that their work contributes to/builds on the existing work in this area by focusing on

	pull rather than push factors. Furthermore, if 'valuing' was a theme (as mentioned in the discussion) why isn't this a theme in the results? For the 'work' theme, they discuss community and education. The difference between this overarching theme and the 'strategies' theme is not clear. For example, if making time for education is important for retention, does this work not depend on organisational support for education (which would fall in your 'strategies' section?) The overlap should be discussed, or if it's not about organisational support but rather about the work of individuals on the ground and not at all about the organisation, that should be clarified. If this theme is more about a positive work environment, maybe the theme needs to be relabelled? Equally, communities feels like it falls in its own category, unless it is discussed in terms of the work people are doing to build a community rather than the experience of the community itself. The examples in each section seem a bit discordant with the themes/aims. On the whole, the examples given throughout this paper are super relevant but the way the data have been categorised limits my ability as a reader to gain insight into what the data *mean.* I feel the theoretical consistency/labelling/discussion of the data is off, limiting the insights/contribution of the work. I discussed much of the above in my original feedback, as well as the need to better situate the findings theoretically (as did the other reviewer) in the discussion. The authors have added some references, but in the results and discussion, the authors have made few substantial changes. I still feel strongly that the authors have conducted a meaningful study that has the potential to contribute to the existing literature- for me, the main priorities are that 1) From the introduction through to the discussion, the unique contributions of the paper need to be highlighted and the messaging needs to be consistent. If the focus is pull factors, the first theme needs a re-think. Regardless, the other themes need to be clarified/theoretically consistent. I have provided my main concerns/ideas above and in my original feedback. 2) In both the introduction and discussion, the authors should better situate their work in the existing literature in this area, demonstrating their theoretical and practical understanding of these issues. References have been added to both the introduction and discussion, but they are mentioned in passing and need to be discussed. This will help demonstrate the relevance of these existing works and how the current work builds on this foundation, so that the authors' own contribution can shine through.<br><br>Ultimately, I feel further revision is required prior to publication to enhance the paper's quality and methodological rigour. I hope the authors have found these comments helpful/constructive and that they continue to iterate this manuscript.

**REVIEWER NAME**	Zhao, Yingxi
**REVIEWER AFFILIATION**	University of Oxford Medical Sciences Division, Nuffield Department of Medicine
**REVIEWER CONFLICT OF INTEREST**	Nothing to declare
**DATE REVIEW RETURNED**	24-Jul-2024

**GENERAL COMMENTS**	I am pleased with the changes and recommend acceptance at its current form.

VERSION 2 – AUTHOR RESPONSE

Reviewer: 1

Dr. Natalie Sanford, KCL

Comments to the Author:

Many thanks to the authors for this revised manuscript. Significant improvements have been made to the abstract and methods sections in response to the reviewer and editor comments. The added supplementary materials are also very helpful. I still feel that additional revision is required prior to publication, as follows:

Introduction:

Although there have been substantial improvements to the introduction, I still feel that the argument/justification for the research could be enhanced and presented more clearly. There are some sections that remain particularly unclear- for instance, what do the authors mean by 'retain' referring to a place as well as an action? As the authors point out in their response letter, there is a limited word count, but I think the points could still be articulated more clearly whilst maintaining the same succinctness. If necessary, I would cut words later on (about insider/outsider positionality, (or make this more succinct/one sentence long); about interview length (could be added to table), or by focusing the discussion) to enable additional insightful discussion of the literature in both the introduction and discussion sections of the manuscript.

The introduction has been edited throughout for clarity and to strengthen the rationale for our approach. In particular, we have developed the paragraph on defining retention to indicate some of the difficulties and justify our chosen definition.

Methods:

The methods section of the paper is much improved. A few comments: What is meant by conceptual rigour? The concept of data saturation is quite contentious in qualitative research; I would delete this sentence. Please ensure consistent verb tense throughout the methods section. Additional detail has been added to both the data collection and analysis sections, which has enhanced methodological consistency and transparency.

We agree about the contentiousness of all the terms used to decide when to stop collecting data. We feel obliged to include something in the manuscript that aligns with our pragmatic approach to theoretical saturation as it is part of the SRQP checklist and we believe readers expect to see something pertaining to it. We have left our reference to Low who does a good job of working through the problems (albeit in a grounded theory sense) and refers to both data saturation and conceptual rigor but have moved to the more commonly used term of theoretical saturation.

We have aligned the tenses for this section.

Results and Discussion:

The authors have incorporated a new sentence or two at the start of each results section which is helpful in better explaining the themes. Still, I find the 'themes' confusing and inconsistent- it seems like they are micro/meso/macro influences of retention (though not discussed as such) and I find the word choice of 'materialities' 'work' and 'strategies' of retention confusing. The first theme, materialities, seems to refer to the on the ground decisions team members make/work arounds they use to mitigate the impact of their work environment? As discussed in my original feedback, it seems to me that these workarounds, whilst propping up the system, would also indicate a need for work-system re-design and may actually threaten retention. The authors also make this argument to a certain extent, which is great, but to me the problem is that this theme seems to directly contrast with their aims/assertion that they make early on in the paper that their work contributes to/builds on the existing work in this area by focusing on pull rather than push factors.

We chose not to orient themes in terms of micro/meso/macro as this may give the impression of a multi-scalar ethnography. Similarly, we do not use the terms push and pull factors as while they are helpful in certain contexts we find we risk being reductive with our data. Our aim to focus on retention is in the context of exodus and an environment that is conducive to leaving, therefore we see no contradiction when our initial theme describes behaviours to facilitate retention in the context of an environment conducive to exodus. It may be that by addressing the name of the theme that this confusion has been allayed.

The theme name was developed as it was greatly influenced sociological literature around materialities. However, descriptions of the development of the theme too this level of detail are inappropriate for this paper. We like your suggestion of the term workarounds and believe it reorients the theme to make it clearer for the reader. We have also made the distinction between workaround and retention work clearer in the manuscript.

Furthermore, if 'valuing' was a theme (as mentioned in the discussion) why isn't this a theme in the results?

We have rewritten the sentence to clarify that valuing was part of several themes rather than one that stands alone.

For the 'work' theme, they discuss community and education. The difference between this overarching theme and the 'strategies' theme is not clear. For example, if making time for education is important for retention, does this work not depend on organisational support for education (which would fall in your 'strategies' section?) The overlap should be discussed, or if it's not about organisational support but rather about the work of individuals on the ground and not at all about the organisation, that

should be clarified. If this theme is more about a positive work environment, maybe the theme needs to be relabelled?

We have added greater scaffolding to clarify that retention work is the day-to-day practices incorporated into the mundane workings of the emergency departments and strategies are more aligned to decisions that participants have made about how to structure their career. Retention work is visible in the day-to-day, strategies less so. This distinction has been added to the results and discussion.

Equally, communities feels like it falls in its own category, unless it is discussed in terms of the work people are doing to build a community rather than the experience of the community itself. The examples in each section seem a bit discordant with the themes/aims. On the whole, the examples given throughout this paper are super relevant but the way the data have been categorised limits my ability as a reader to gain insight into what the data *mean.* I feel the theoretical consistency/labelling/discussion of the data is off, limiting the insights/contribution of the work.

Exactly, it is the work of building the community that the theme refers to. Some of the interview data supports why this is important but this is well established. The day-to-day and effortful nature of building teams is what is important. We have re-incorporated the term retention work into the subheading to try and clarify this and make the theme more consistent.

I discussed much of the above in my original feedback, as well as the need to better situate the findings theoretically (as did the other reviewer) in the discussion. The authors have added some references, but in the results and discussion, the authors have made few substantial changes. I still feel strongly that the authors have conducted a meaningful study that has the potential to contribute to the existing literature- for me, the main priorities are that 1) From the introduction through to the discussion, the unique contributions of the paper need to be highlighted and the messaging needs to be consistent. If the focus is pull factors, the first theme needs a re-think. Regardless, the other themes need to be clarified/theoretically consistent. I have provided my main concerns/ideas above and in my original feedback.

We have renamed the first theme and added scaffolding and clarifications around the other themes which we hope adds consistency to our arguments.

2) In both the introduction and discussion, the authors should better situate their work in the existing literature in this area, demonstrating their theoretical and practical understanding of these issues. References have been added to both the introduction and discussion, but they are mentioned in passing and need to be discussed. This will help demonstrate the relevance of these existing works and how the current work builds on this foundation, so that the authors' own contribution can shine through.

We have reviewed our discussion in relation to the BMJ Open guidelines on writing a discussion, providing a statement of the principal findings; strengths and weaknesses of the study; strengths and weaknesses in relation to other studies, discussing important differences in results; the meaning of the study: possible explanations and implications for clinicians and policymakers; and unanswered questions and future research.

The literature we draw on here is integrated into the points we make about the contribution of the paper, and it is difficult to expand further while maintaining conciseness of the overall paper.

Ultimately, I feel further revision is required prior to publication to enhance the paper's quality and methodological rigour. I hope the authors have found these comments helpful/constructive and that they continue to iterate this manuscript.

Reviewer: 2

Dr. Yingxi Zhao, University of Oxford Medical Sciences Division

Comments to the Author:

I am pleased with the changes and recommend acceptance at its current form.

VERSION 3 - REVIEW

**REVIEWER NAME**	Sanford, Natalie
**REVIEWER AFFILIATION**	KCL
**REVIEWER CONFLICT OF INTEREST**	None to declare
**DATE REVIEW RETURNED**	27-Aug-2024

**GENERAL COMMENTS**	Many thanks to the authors for their thoughtful responses to my comments. The changes to the introduction and small changes to the results/discussion have really improved the clarity of the findings. I think that the manuscript is well articulated, clearly answers the research question, and is a valuable contribution to the literature. I look forward to seeing it in print!
